# Nanoparticle contrast‐enhanced computed tomography and magnetic resonance imaging of vascularization of a subcutaneous niche for islet transplantation

**DOI:** 10.1002/btm2.10740

**Published:** 2024-12-13

**Authors:** Simone Capuani, Jocelyn Nikita Campa‐Carranza, Nathanael Hernandez, Renuka T. R. Menon, Rohan Bhavane, Gabrielle E. Rome, Laxman Devkota, Ketan B. Ghaghada, Ananth V. Annapragada, Corrine Ying Xuan Chua, Andrew A. Badachhape, Alessandro Grattoni

**Affiliations:** ^1^ Department of Nanomedicine Houston Methodist Research Institute Houston Texas USA; ^2^ School of Medicine and Health Sciences, Tecnologico de Monterrey Monterrey NL Mexico; ^3^ Department of Medicine‐Endocrinology Baylor College of Medicine Houston Texas USA; ^4^ Department of Radiology Texas Children's Hospital Houston Texas USA; ^5^ Department of Radiology Baylor College of Medicine Houston Texas USA; ^6^ Department of Obstetrics and Gynecology Baylor College of Medicine Houston Texas USA; ^7^ Department of Surgery Houston Methodist Hospital Houston Texas USA; ^8^ Department of Radiation Oncology Houston Methodist Hospital Houston Texas USA

**Keywords:** cell encapsulation, computed tomography, contrast‐enhanced imaging, islet transplantation, magnetic resonance imaging, revascularization, T2‐mapping

## Abstract

Revascularization plays a critical role in the successful engraftment of transplanted pancreatic islets, which are inherently rich in capillaries to meet their high metabolic demands. Innovative islet encapsulation strategies such as the NICHE (neovascularized implantable cell homing and encapsulation), generate a prevascularized transplantation site that allows for direct integration of the graft with the systemic circulation. Timing the transplantation is key to maximizing islet engraftment and survival, especially in diabetic individuals, who exhibit impaired wound healing. Therefore, in this study, we explored different methods to assess vascular development within NICHE in vivo in a non‐invasive fashion. We effectively tracked neoangiogenesis using nanoparticle contrast‐enhanced computed tomography (nCECT), observing a steady increase in vascularization over an 8‐week period, which was confirmed histologically. Next, we estimated relative vascularization changes via T2 mapping with magnetic resonance imaging (MRI) before and after islet transplantation. On the first day post‐transplantation, we measured a slight decrease in T2 values followed by a significant increase by day 14 attributable to islet revascularization. Our findings underscore the potential of non‐invasive imaging techniques to provide insightful information on the readiness of the transplant site within cell encapsulation systems to support cell graft transplantation.

**Translational Impact Statement**

Emerging islet encapsulation technologies for the treatment of type 1 diabetes require prevascularization of the transplant site. Impaired wound healing in diabetic patients alters angiogenesis, therefore assessing the vascularization of the transplant site ahead of the transplant is critical to maximize islet engraftment and therapeutic outcomes. Herein we present and validate non‐invasive imaging techniques that provide insightful information on the vascular development within a cell encapsulation platform with direct vascularization.

## 1 INTRODUCTION

Revascularization of pancreatic islets post‐transplantation is critical for their successful engraftment.^1^ In their native state, islets are densely interwoven with capillaries that deliver the oxygen and nutrients needed to support their high metabolic activity.^2^ Despite constituting only 1% of the total pancreas volume, islets receive up to 20% of its blood supply,^3^ highlighting the importance of adequate vascularization. The lack of revascularization contributes significantly to the challenges faced in clinical islet transplantation. Currently, the standard clinical approach involves infusing of pancreatic islets, isolated from a cadaveric donor, into the portal vein of a diabetic patient.^4^ While effective, this method requires systemic immunosuppression to prevent rejection and a substantial dose of islets to achieve euglycemia, as 50%–70% of the transplanted islets fail to engraft.^5^, ^6^, ^7^ To address these issues, semipermeable implantable pouches are used to encapsulate islets or stem cell‐derived insulin‐producing cells.^8^, ^9^ These pouches are made of semipermeable membranes that physically block immune cell infiltration while allowing the passive diffusion of oxygen and nutrients. However, they also prevent the direct revascularization, leading to hypoxia and cell death, a scenario worsened by the fibrotic encapsulation of the implant.^10^, ^11^

To overcome these limitations, islet encapsulation platforms that enable direct revascularization are gaining attention.^12^ These approaches involve simultaneous delivery of implants and islets, or preconditioning and prevascularizing the transplant site. The former strategy has shown promising results in preclinical models,^13^, ^14^, ^15^ and moderate improvements in glycemic management in clinical trials (NCT03162926 and NCT03163511).^16^, ^17^ Prevascularizing the transplant site before islet delivery has also resulted in successful engraftment and revascularization in preclinical and clinical studies. For instance, Pepper et al. exploited the foreign body response to a temporary implanted catheter to prevascularize a subcutaneous site for subsequent islet transplantation in mice.^18^ Other groups have used similar methods, where spaceholders were embedded or coated in angiogenic materials.^19^, ^20^, ^21^, ^22^ An analogous strategy is being evaluated by Sernova with its Cell Pouch in a phase I/II clinical trial (NCT03513939). These studies achieved encouraging results in ameliorating islet engraftment and long‐term diabetes management. However, direct vascularization leaves the graft exposed to the immune system, requiring chronic systemic immunosuppression to prevent rejection.

Our group developed a subcutaneous implant called NICHE (neovascularized implantable cell homing and encapsulation), which combines a vascularized cell compartment with localized immunosuppressant delivery from a refillable drug reservoir.^23^, ^24^ NICHE achieved complete diabetes reversal in an allogeneic rat model, confining immunosuppressive drugs to the device site and protecting the graft from rejection without systemic effects.^25^, ^26^ Prevascularization is enhanced by pre‐loading the NICHE cell reservoir with mesenchymal stem cells (MSCs), known for their angiogenic and immunomodulatory properties.^27^, ^28^, ^29^

The timing of the prevascularization phase is crucial for optimal islet engraftment, especially in type 1 diabetes patients who have impaired wound healing. Importantly, the foreign body response and wound healing mechanisms responsible for generating a vascularized site can vary among individuals.^30^ Therefore, assessing prevascularization level before transplantation can maximize graft survival and function, reducing the islet dose needed for euglycemia.

In this study, we aimed to define and validate methodologies to track and assess in vivo neoangiogenesis in a cell encapsulation platform with direct vascularization. Using the NICHE platform, we monitored neovasculature formation over 8 weeks via nanoparticle contrast‐enhanced computed tomography (nCECT). We used liposomal‐based iodine nanoparticles (Lip‐I) as contrast agents for nCECT. Importantly, these agents, developed and extensively validated,^31^, ^32^, ^33^, ^34^ exhibit long‐circulating properties (~14 h half‐life), making them ideal for detailed vascular architecture and morphometric analyses.^35^ The imaging data provided a fractional blood volume (FBV) within NICHE, correlated to histological evidence. Additionally, we used MRI to estimate vascularization changes before and after islet transplantation at different times post‐device implantation with T2 mapping.

## 2 MATERIALS AND METHODS

### 2.1 NICHE device

The fabrication of NICHE followed previously established protocols.^24^, ^25^ In summary, two polyethersulfone (PES) nanoporous membranes, each with a pore size of 30 nm (Sterlitech), along with two sets of nylon meshes (Elko Filtering), were securely attached to a 3D printed polyamide structure (PA2200, EOS) measuring 30.4 mm × 15.4 mm × 3.8 mm using a silicone‐based adhesive approved for implantation (MED3‐4213, Nusil). This adhesive was also employed in the creation of the silicone ports. To ensure sterility, all components were sterilized in an autoclave before being assembled in a sterile environment under a laminar flow hood. The fully assembled NICHE units underwent a final sterilization process using ethylene oxide gas at the Houston Methodist Research Institute (HMRI) in their Current Good Manufacturing Practice (CGMP) core facility.

### 2.2 Animal experiments

In this research, male F344 rats aged 8 weeks (Charles River, Houston, TX, USA) were utilized. The care and use of these animals adhered to the guidelines set by the American Association for Laboratory Animal Science. The rats were housed at the Houston Methodist Research Institute (HMRI) animal facility and the Texas Children's Hospital Feigin Center animal vivarium, with the research activities taking place under the auspices of the HMRI Comparative Medicine Program (CMP). These activities were conducted in compliance with the Animal Welfare Act, the Public Health Service (PHS) Animal Welfare Policy, and the NIH Guide for the Care and Use of Laboratory Animals' principles. The Institutional Animal Care and Use Committee (IACUC) at HMRI and Baylor College of Medicine, approved the humane use of animals in this research, as well as all procedures outlined in the IACUC protocol number IS00007362 (HMRI) and AN‐8907 (BCM). The animals were kept in standard conditions, with unrestricted access to water and a conventional lab diet.

### 2.3 NICHE deployment

The cell reservoir of the sterile NICHE was populated with mesenchymal stem cells (MSCs) to promote vascularization and tissue development within the reservoir, following methods outlined in our earlier work.^24^, ^25^ In summary, MSCs harvested from F344 rat bone marrow (RAFMX‐01001, Cyagen, Lot. 210330H61) were suspended in a hydrogel composed of pluronic F‐127 (20% PF‐127 in DMEM, Sigma). This suspension was then delivered into the NICHE cell reservoir through its central silicone port, with each NICHE receiving approximately 5 × 10^5^ MSCs. Following the injection, the MSC‐laden NICHEs were surgically implanted into a subcutaneous pocket, which was created by making a 2 cm long incision on the back of the rat, employing techniques detailed in our previous publications.^24^, ^25^

### 2.4 Nanoparticle contrast‐enhanced computed tomography

Computed tomography (CT) scans were conducted using a Siemens Inveon small animal micro‐CT system. For sedation, animals received an initial dose of 3% isoflurane, were positioned on the CT scanner's bed, and then the isoflurane concentration was adjusted to between 2% and 2.5% for maintenance, administered through a nose cone. The CT scan settings included an 80 kV voltage, 0.5 mA current, 240 ms X‐ray exposure time, 540 projections, and an isotropic spatial resolution of 107 μm, with a total scan duration of approximately 20 min. For nCECT imaging, a long‐circulating liposomal‐iodine (Lip‐I) contrast agent was used.^34^, ^36^, ^37^ The protocol involved an initial scan before contrast administration, the intravenous injection of the Lip‐I contrast agent at a dosage of 1 g iodine per kilogram of body weight via the tail vein, and then a subsequent scan. Data sets were calibrated in Hounsfield Units (HU) for analysis, which was conducted using Osirix medical imaging software (version 5.8.5, 64‐bit, Pixmeo). The FBV derived from CT was determined by calculating the ratio of signal enhancement in the tumor to that in the blood (specifically, in the inferior vena cava, IVC) comparing delayed nCECT scans to immediate nCECT scans, according to the following equation^32^, ^36^, ^38^, ^39^:

(1) $$\mathrm{FBV}=\frac{HU_{N,\text{acute}}-HU_{N,\text{delay}}}{HU_{\mathrm{IVC},\text{acute}}-HU_{\mathrm{IVC},\text{delay}}}$$

where HU_N_ indicates mean CT signal (expressed in HU) for segmented NICHE cell reservoir volume and HU_IVC_ indicates mean CT signal for region of interest (ROI) drawn in the IVC.

### 2.5 In vivo vascularization assessment via nCECT

Rats (*n* = 12) were implanted with NICHE devices loaded with MSCs as described earlier. Groups of animals were imaged via nCECT at 2, 3, 4, 6, and 8 weeks post‐implantation to assess vascular development via FBV estimates. After imaging, at 4, and 8 weeks, *n* = 4 rats/timepoint were euthanized and the devices and surrounding subcutaneous tissue were collected en bloc and fixed in 10% buffered formalin. Another group of *n* = 4 animals was euthanized at 6 weeks post‐implantation and perfused with a radiopaque silicone rubber injection compound (Microfil, Flow Tech Inc.) according to a published protocol.^40^ Briefly, rats were deeply sedated with 3.5% isoflurane and their thoracic cavity was opened to expose the heart. Heparin sodium (1500 USP, JT Baker) was injected into the left ventricle using a syringe with a 25G needle. Next, an 18G blunt needle was forced through the opening previously created in the left ventricle and pushed out into the aortic arc, where it was clamped with a hemostat. The needle was connected to a peristaltic pump through tubing and 250 mL of warm (37°C) sterile saline solution was perfused after severing the right atrium, euthanizing the animal by exsanguination. Then, 30 mL of Microfil solution was perfused, the needle was removed from the hearth and the carcass was placed at 4°C overnight to allow the silicone solution to cure. The NICHE device was explanted, imaged via CT at a higher resolution (isotropic spatial resolution of 35 μm) and subsequently placed in 10% buffered formalin for fixation. CT image acquisition parameters were: 80 kVp, 0.5 mA, 850 ms X‐ray exposure, 540 projections, 35 μm isdotropic spatial resolution, scan time ~ 20 min.

### 2.6 Histology and immunohistochemistry analysis

Formalin‐fixed samples were dehydrated in standard ethanol and NICHE polyamide structure was removed to allow sectioning. The samples were washed in xylene, embedded in paraffin and sectioned (5 μm). Hematoxylin–eosin (H&E) staining was performed on sections at the HMRI Research Pathology Core. To label blood vessels, tissue sections of explanted NICHE were stained with B. simplicifolia lectin (L2140, Sigma, 1:100). All sections were imaged with an automated microscope (BZ‐X810, Keyence).

H&E‐stained sections were scanned at 4× magnification while for each lectin‐stained section, *n* = 10 ROIs were acquired at 40× magnification. Lectin‐stained capillaries were measured manually using QuPath 0.5.0 and capillary density and capillary area fraction were calculated according to the following equations:

(2) $$\begin{array}{c} \text{Capillary density}=\frac{\text{Number of capillaries}}{\text{Total area of}\;\mathrm{ROI}} \end{array}$$

(3) $$\begin{array}{c} \text{Capillary area fraction}=\frac{\text{Area occupied}\;\mathrm{by}\;\text{capillaries}}{\text{Total area of}\;\mathrm{ROI}}\times 100 \end{array}$$

### 2.7 Pancreatic islet isolation and transplantation

Pancreatic islets from syngeneic sources were procured from *n* = 40 F344 donor rats, employing a methodology outlined in prior research.^25^ Initially, donor rats were humanely euthanized through an overdose of isoflurane. The pancreatic duct was then cannulated for perfusion with 9 mL of CIzyme RI collagenase (Vitacyte) and 0.2 μg/mL DNAse (dornase alfa; Genentech) in a Hanks balanced salt solution (HBSS; Gibco) enriched with 10 mm HEPES (Gibco). Subsequently, the pancreas was excised and subjected to a 20‐min digestion in the aforementioned collagenase mixture. The enzymatic reaction was halted with 20% fetal bovine serum (FBS, Gibco), followed by mechanical dissociation of the tissue. The material was then filtered through a 500 μm mesh screen, and the islets were separated using an OptiPrep density gradient (Sigma). After cultivating the islets for 3 days, they were encased in a thermoresponsive collagen hydrogel (Advanced Biomatrix) and introduced into the NICHE transcutaneously via the central silicone port (10,000 IEQ/kg).

### 2.8 Magnetic resonance imaging—T2 mapping

MR imaging was performed on a 1 T permanent scanner (M7 system, Aspect Imaging, Shoham, Israel). Animal sedation was induced at 2.5% isoflurane in a separate induction box, and the animals were then setup on the MRI bed and maintained at 1%–1.5% isoflurane via a nose cone setup built into the animal bed. Respiration was monitored by a pneumatic pressure pad placed underneath the animal abdomen. Warm water was circulated underneath the animal bed to maintain animal temperature. Animals underwent a spin‐echo, T2 mapping sequence^41^ with the following parameters: TR: 2500 ms, TE: 15, 30, 45, and 60 ms, FOV: 60 mm, matrix: 120 × 120, and acquisition time: 22 min (~5.5 min/TE). The resulting maps had 500 micron isotropic voxel resolution with a field of view centered on the NICHE device.

### 2.9 In vivo vascularization assessment via MRI

Rats (*n* = 8) were implanted with NICHE loaded with MSCs as described earlier. The animals were divided into two groups (*n* = 4/group), which received a syngeneic pancreatic islets transplant at 4 and 6 weeks post‐implantation respectively. MRI was performed the day before and after islet transplantation, and again at 2 weeks post‐transplantation. Then, the animals were euthanized and the NICHE device was collected and fixed in 10% buffered formalin.

### 2.10 Tissue clarification and lightsheet microscopy

One rat was implanted with a NICHE device loaded with MSCs as previously described. After 6 weeks, 10,000 IEQ/kg were transplanted in the NICHE cell reservoir. Two weeks later, the animal was anesthesized and received a tail vein injection of Heparin (0.5 mL at 15 mg/mL, J.T. Baker) and Lectin‐DyLight649 (1 mL at 1 mg/mL, Vector Laboratories). Next, the rat was euthanized by transcardiac perfusion of PBS 1× followed by 4% paraformaldehyde. The NICHE device was explanted and placed in paraformaldehyde overnight. The cell reservoir tissue was then extracted, clarified, and stained using the EZ clear protocol.^42^ Briefly, the tissue was delipidated in tetrahydrofuran and washed in PBS 1×. For insulin staining, the sample was submerged in 5 mL blocking buffer (PBS 1× with 0.08% Triton X‐100, 2% goat serum, and 0.05% sodium azide) for 48 h. It was then incubated in a 3 mL solution of the primary antibody (Rb anti‐rat Insulin, Cell Signaling, 1:200 in blocking buffer) for 4 days, followed by three 2‐h washes in PBS 1×. Subsequently, the tissue was incubated in 2 mL of the secondary antibody solution (goat anti‐Rb AF555, Invitrogen, 1:200 in blocking buffer) for 4 days. Finally, the sample was washed in PBS 1× three times, incubated overnight in PBS 1× with 0.05% sodium azide, and placed in EZ view solution until equilibrated.

For imaging, the equilibrated sample was embedded in a 1% agarose hydrogel and mounted on a custom‐designed sample holder.^42^ The sample was placed in a custom‐made chamber containing EZ view solution and imaged using a Zeiss Lightsheet Z.1 microscope. Data were acquired with a 5× lens at 0.5× zoom, with a resolution of 1.829 μm × 1.829 μm × 7.03 μm (*X*:*Y*:*Z*) in a tiled Z‐stack sequence with 20% overlap between tiles. Lectin‐DyLight649 fluorescence was captured by exciting the sample with a 638 nm laser at 10% power and a 300 ms exposure time, while Insulin‐AF555 fluorescence was captured with a 561 nm laser at 5% power and a 30 ms exposure time. The dataset was stitched and aligned using Stitchy (Translucence Biosystems), and visualization was performed using Imaris (Oxford Instruments).

### 2.11 Statistical analysis

Statistical analysis was performed using Graphpad Prism 9.3.1 using one‐way ANOVA (*p < 0.05, **p < 0.01, ***p < 0.001, and ****p < 0.0001).

## 3 RESULTS AND DISCUSSION

### 3.1 Nanoparticle contrast‐enhanced CT imaging of NICHE

**FIGURE 1:**
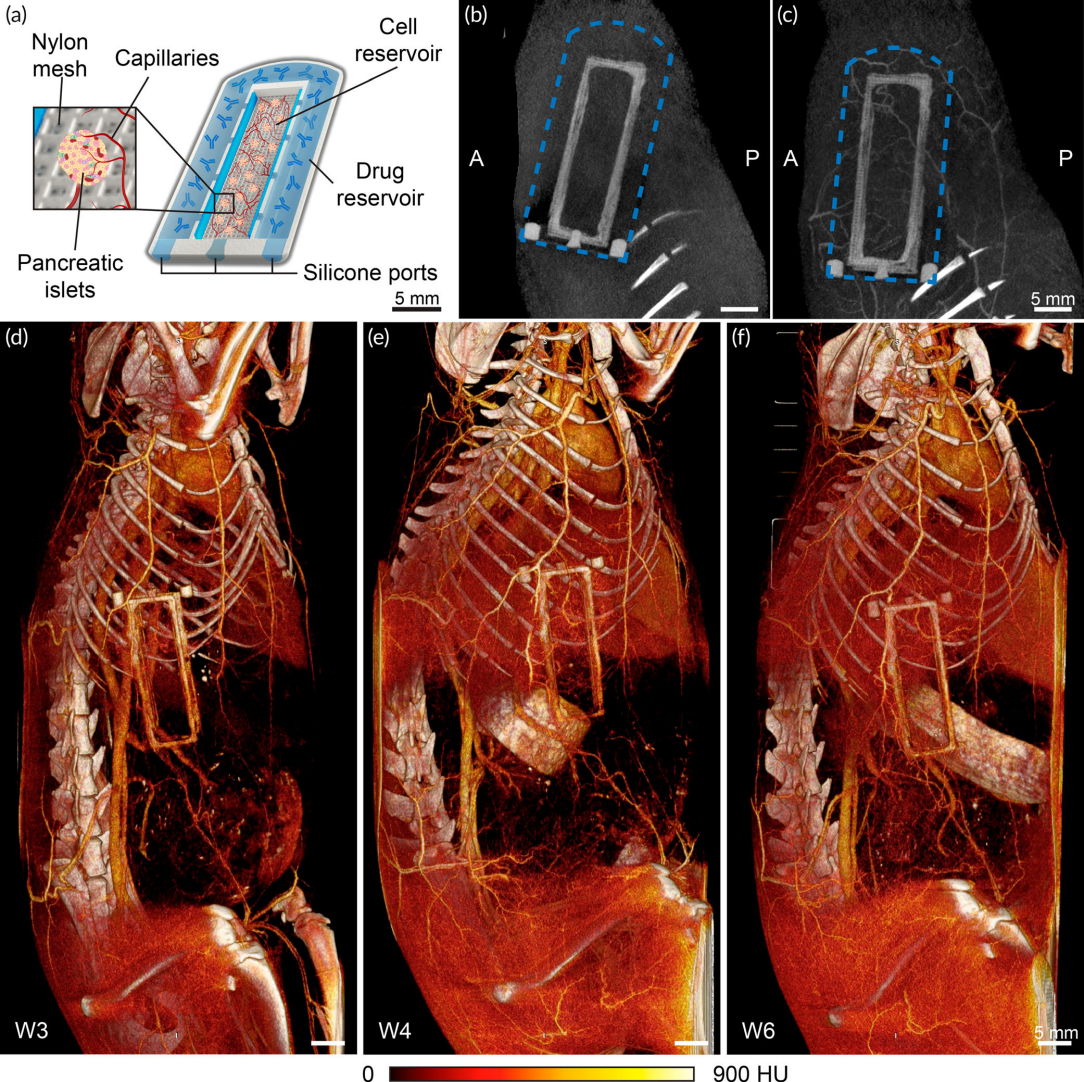
Liposomal contrast efficacy assessment. (a) Diagram of the NICHE platform. Scale bar 5 mm. (b) CT image of NICHE prior to Lip‐I contrast injection. Scale bar 5 mm. (c) CT image of NICHE post Lip‐I contrast injection. Whole body high‐resolution 3D volume rendered nCECT images of rats implanted with NICHE at (d) 3 weeks, (e) 4 weeks and (f) 6 weeks post implantation. Scale bar 5 mm.

NICHE is a subcutaneously implantable cell encapsulation platform comprising non‐biodegradable materials and is intended for long‐term administration.^24^, ^25^ The device features local immunosuppression and a vascularized compartment for pancreatic islet transplantation (Figure 1a) in the context of treating type 1 diabetes. The growth of arterioles and capillaries within the NICHE's cell reservoir is driven by the presence of MSCs, creating a dense capillary network to enhance oxygen delivery and maximize the engraftment of transplanted islets. Our previous studies^24^, ^25^, ^27^ have shown that extensive vascularization within NICHE occurs within 4–6 weeks post‐implantation in healthy animals, which was identified as the optimal window for islet transplantation. However, in diabetic subjects, impaired wound healing can delay the vascularization of the cell reservoir, reducing the transplant's engraftment potential.^30^, ^43^ Here, we evaluate the potential of contrast‐enhanced imaging to monitor the vascularization of the NICHE platform. The NICHE device is detectable via CT imaging due to the radiopaque properties of the silicone used to attach the nylon meshes on the 3D‐printed structure and fabricate the refilling plugs (Figure 1b). Following the intravenous injection of Lip‐I contrast agent, nCECT images revealed a vascular network surrounding and infiltrating the implant (Figure 1c). Additionally, whole body 3D volume renderings show the development and enlargement of afferent blood vessels over time (Figure 1d–f). These findings demonstrate the utility of nCECT for in vivo assessment of vascular development, and indicate that the blood vessels within the device are integrated with the systemic circulation.

### 3.2 In vivo vascularization assessment via nCECT

**FIGURE 2:**
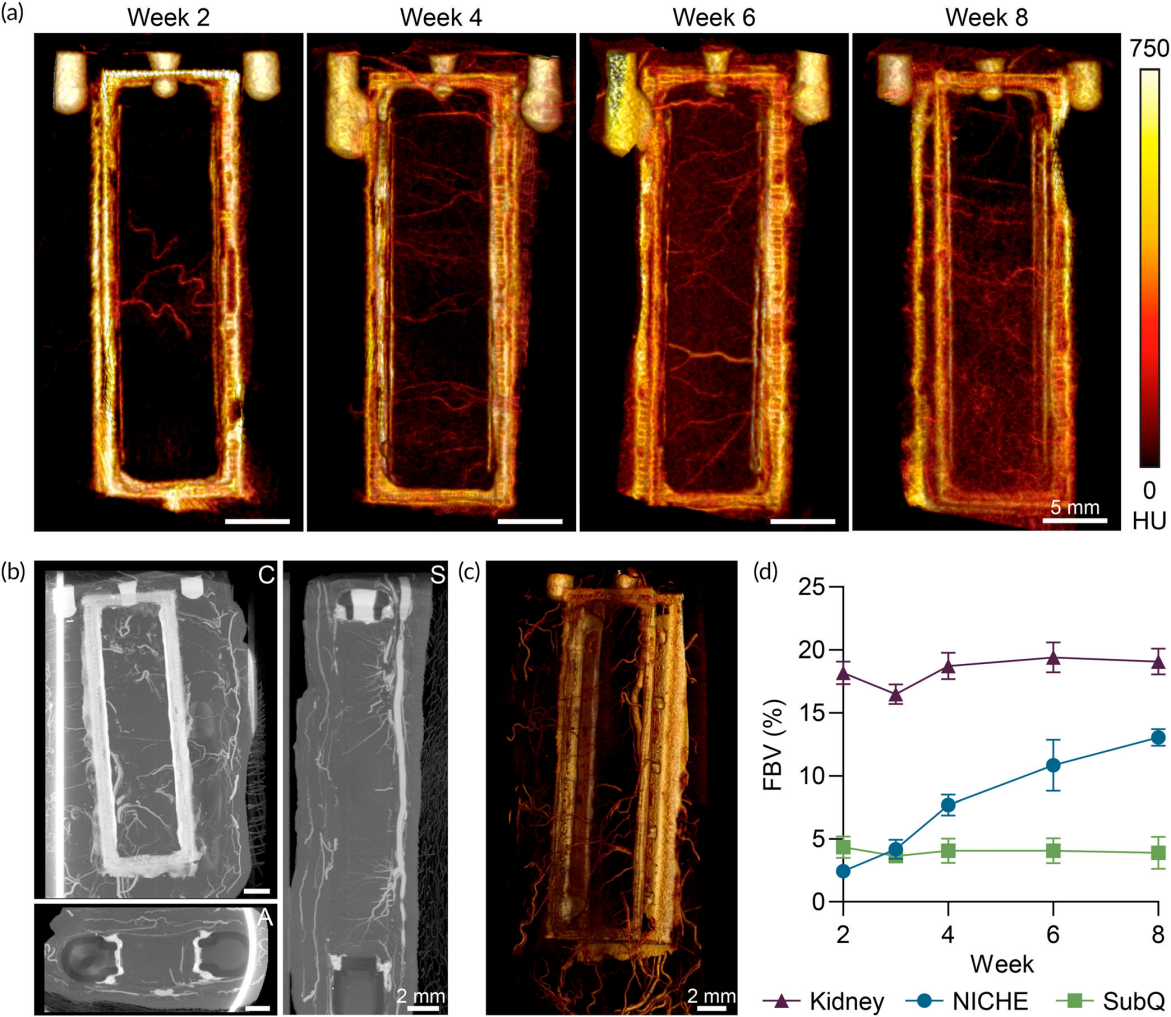
Vascularization development assessment via liposomal contrast‐enhanced CT. (a) 3D volume rendered nCECT images of NICHE implanted in rats at 2, 4, 6 and 8 weeks post implantation. Scale bar 5 mm. Ex vivo high‐resolution. (b) 2D images and (c) 3D rendering of NICHE from animals perfused with Microfil solution. Scale bars 2 mm. (d) nCECT‐derived fractional blood volume (FBV) in NICHE, kidney, and subcutaneous tissue contralateral to NICHE (*n* = 4/timepoint).

To demonstrate the capability of nCECT in tracking vascular development within our implant, we performed a longitudinal study imaging NICHE devices implanted in rats over an 8‐week period. The 3D volume‐rendered nCECT images illustrate the increasing number of arterioles and capillaries in the NICHE over time (Figure 2a). Interestingly, ex vivo high‐resolution Microfil‐enhanced CT revealed a large artery running along the device surface, branching and penetrating the cell reservoir (Figure 2b), consistent with observations from whole body images (Figure 1d–f). Additionally, numerous arterioles are visible surrounding the implant (Figure 2c), suggesting robust integration with the systemic vascular network.

We quantitatively assessed capillary ingrowth in the NICHE cell reservoir by measuring the FBV. The longitudinal analysis showed a steady increase in FBV within the NICHE over time, surpassing the FBV in the contralateral subcutaneous space by week 4 post‐implantation and tripled by week 8 (Figure 2d). However, the NICHE does not achieve the FBV observed in the kidney, a preferred site for preclinical islet transplantation, due to its high vascular density.^44^, ^45^, ^46^

**FIGURE 3:**
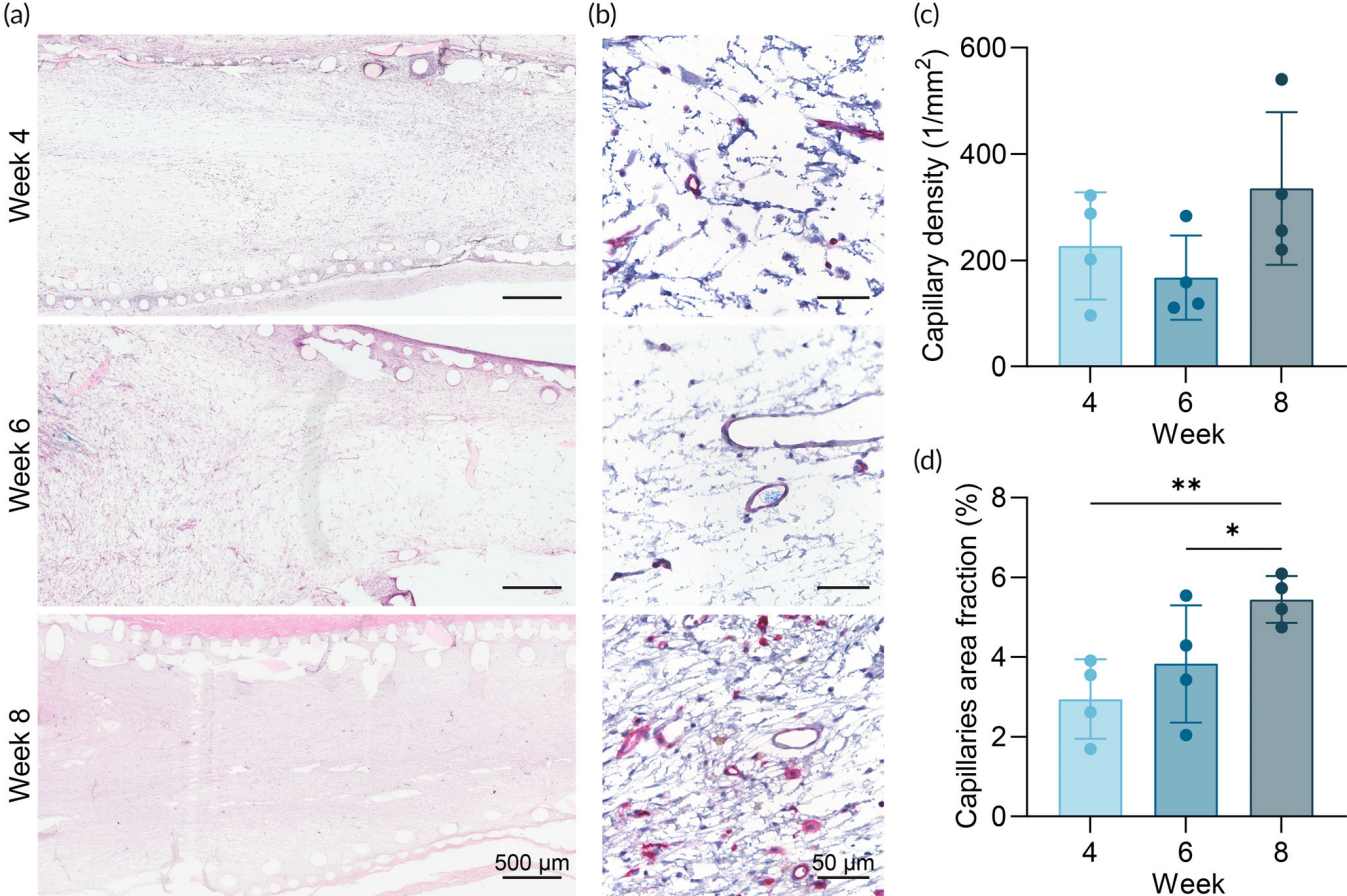
Histological analysis of vascular development. (a) Representative scans of H&E‐stained sections of NICHE explanted at 4, 6, and 8 weeks post‐implantation. Scale bar 500 μm. (b) Representative images of lectin (pink) stained sections of NICHE cell reservoir tissue explanted at 4, 6, and 8 weeks post‐implantation acquired at 40× magnification. Scale bar 50 μm. (c) Capillary density and (d) capillaries fractional area obtained from images of lectin‐stained sections (*n* = 4/group). One‐way ANOVA **p < 0.01.

Histological images reveal the formation of fully vascularized tissue within the NICHE cell reservoir starting at week 4 (Figure 3a,b). Vascular density assessment (Figure 3c) shows stable metrics throughout the timepoints in contrast with the increasing FBV observed (Figure 2d). This discrepancy could be explained by the presence of newly formed, non‐patent vessels at 4 weeks post‐implantation, resulting in lower FBV. In contrast, the capillary area fraction analysis shows a rising trend over time, aligning with the FBV measurements (Figure 3d).

Overall, nCECT appears to be a valid method for in vivo assessment of vascular development in a cell encapsulation platform with direct vascularization, providing insight into the formation and distribution of blood vessels and their integration with the systemic circulation.

### 3.3 In vivo assessment of vascular changes via MRI

**FIGURE 4:**
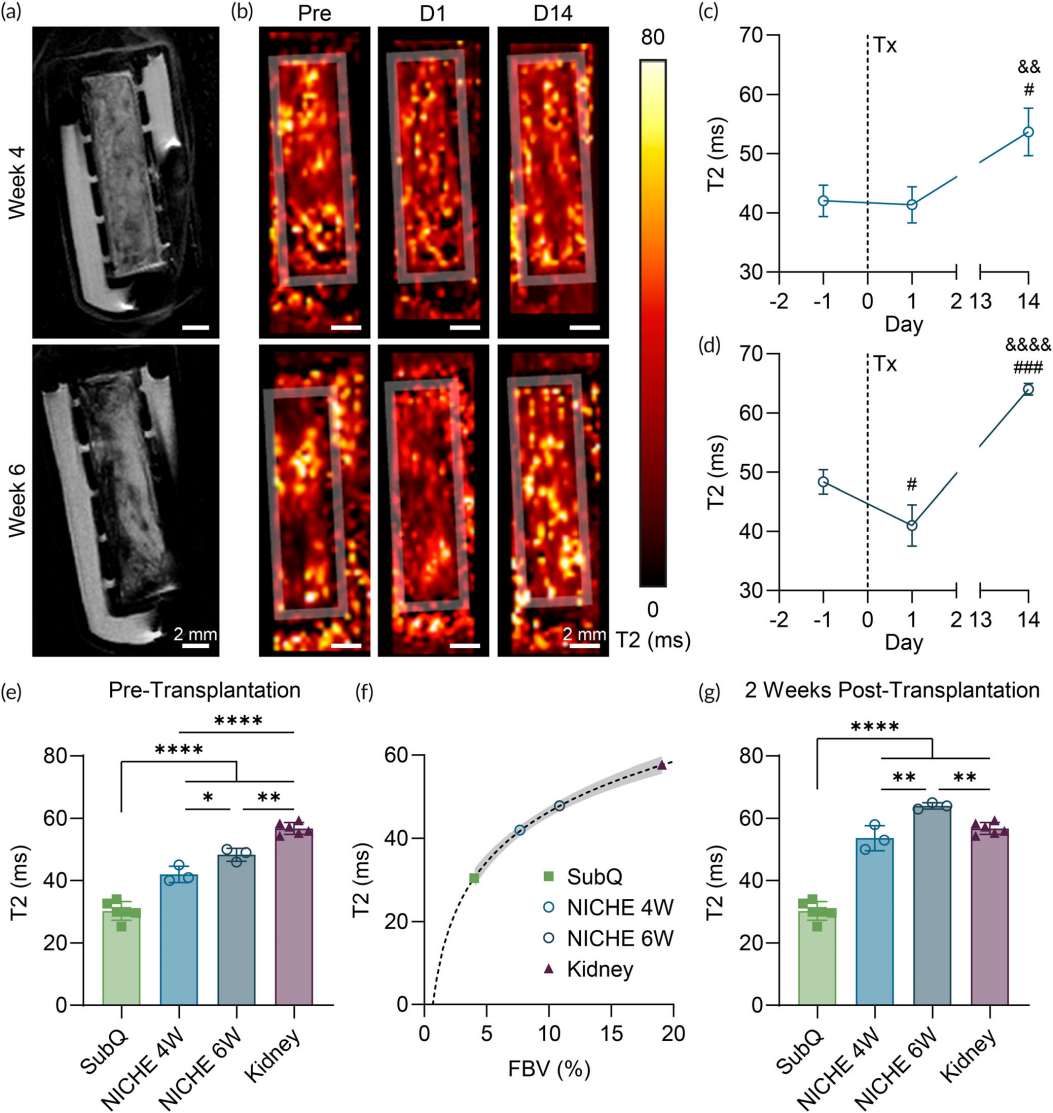
Non‐contrast MRI assessment of T2 values as a measure of vascularization. (a) In vivo MRI images of NICHE devices implanted in rats. Scale bars 2 mm. (b) T2 mapping of the NICHE cell reservoir tissue. Scale bars 2 mm. Average T2 values calculated within NICHE cell reservoir 1 day before, 1 day and 14 days after islet transplantation in NICHE implanted for (c) 4 weeks and (d) 6 weeks (*n* = 3/group). ^#^Comparison versus day −1 and ^&^comparison versus day 1. Dotted line at day 0 indicates the islet transplant. (e) Average T2 values calculated within NICHE cell reservoir, contralateral subcutaneous tissue and kidney 1 day before islet transplantation (*n* = 3–6/group). (f) Correlation between average T2 values obtained via MRI and FBV calculated from CT images. Dotted line represents logarithmic interpolation: T2 = a*log(FBV) + *b*; (*a* = 40.06, *b* = 6.303). Gray area indicates 95% confidence interval. (g) Average T2 values calculated within NICHE cell reservoir, contralateral subcutaneous tissue, and kidney 14 days after islet transplantation (*n* = 3–6/group). One‐way ANOVA *^,#^ *p* < 0.05, **^,&&^ *p* < 0.01, ^###^ *p* < 0.001, ****^,&&&&^ *p* < 0.0001.

A fundamental hurdle to successful islet transplantation is the limited or impaired oxygen delivery that occurs within immune isolating constructs or before graft revascularization in platforms that grant direct access to the vascular network.^12^, ^47^, ^48^ T2 mapping is a common and non‐invasive method of estimating revascularization and oxygen saturation.^49^, ^50^ While we are unable to separate T2 contributions due to oxygen saturation from vascular changes in the current experiment, assuming no changes in oxygen saturation in the timeframe of this experiment we can correlate the T2 with FBV derived from CE‐CT. We employed this technique to provide non‐contrast estimates within the NICHE cell reservoir before and after islet transplantation, performed at 4 or 6 weeks post‐implantation (Figure 4a,b). In the week‐4 group, average T2 values in the cell reservoir remained consistent before and after transplantation, then increased after 14 days (Figure 4c). In contrast, the week‐6 group showed a decrease in T2 values 1 day post‐transplant, followed by a significant increase by day 14 (Figure 4d). The transplantation procedure, involving transcutaneous injection into the cell reservoir, can disrupt part of the existing capillary network. Such disruption is likely more pronounced in a highly vascularized environment, which could explain the initial drop in T2 values in the week‐6 group. The observed increase 2 weeks post‐transplantation in both groups likely reflects graft revascularization and an enhanced blood supply to support islet function.^1^, ^51^, ^52^

We then compared the average T2 values measured in the NICHE 1 day prior to and 2 weeks post‐transplantation with those in the subcutaneous tissue contralateral to the device and in the kidney (Figure 4e,g). The NICHE and the kidney groups had significantly higher T2 values than the subcutaneous tissue in both instances. Before transplantation, NICHEs in the week‐6 group exhibited higher T2 values than those in the week‐4 group, though still below kidney levels, consistent with FBV measurements (Figure 2d). Notably, T2 values in the NICHE 2 weeks post‐transplantation were similar to (week‐4 group) or higher than (week‐6 group) those in the kidney, supporting the hypothesis of increased vascularization induced by the graft.

Lastly, we compared average T2 mapping estimates before islet transplantation with FBV assessments from nCECT (Figure 4f). Despite being conducted in separate animal cohorts, the two measurements showed a strong logarithmic correlation (*R* ^2^ = 0.9983), providing further evidence of neovascularization.

**FIGURE 5:**
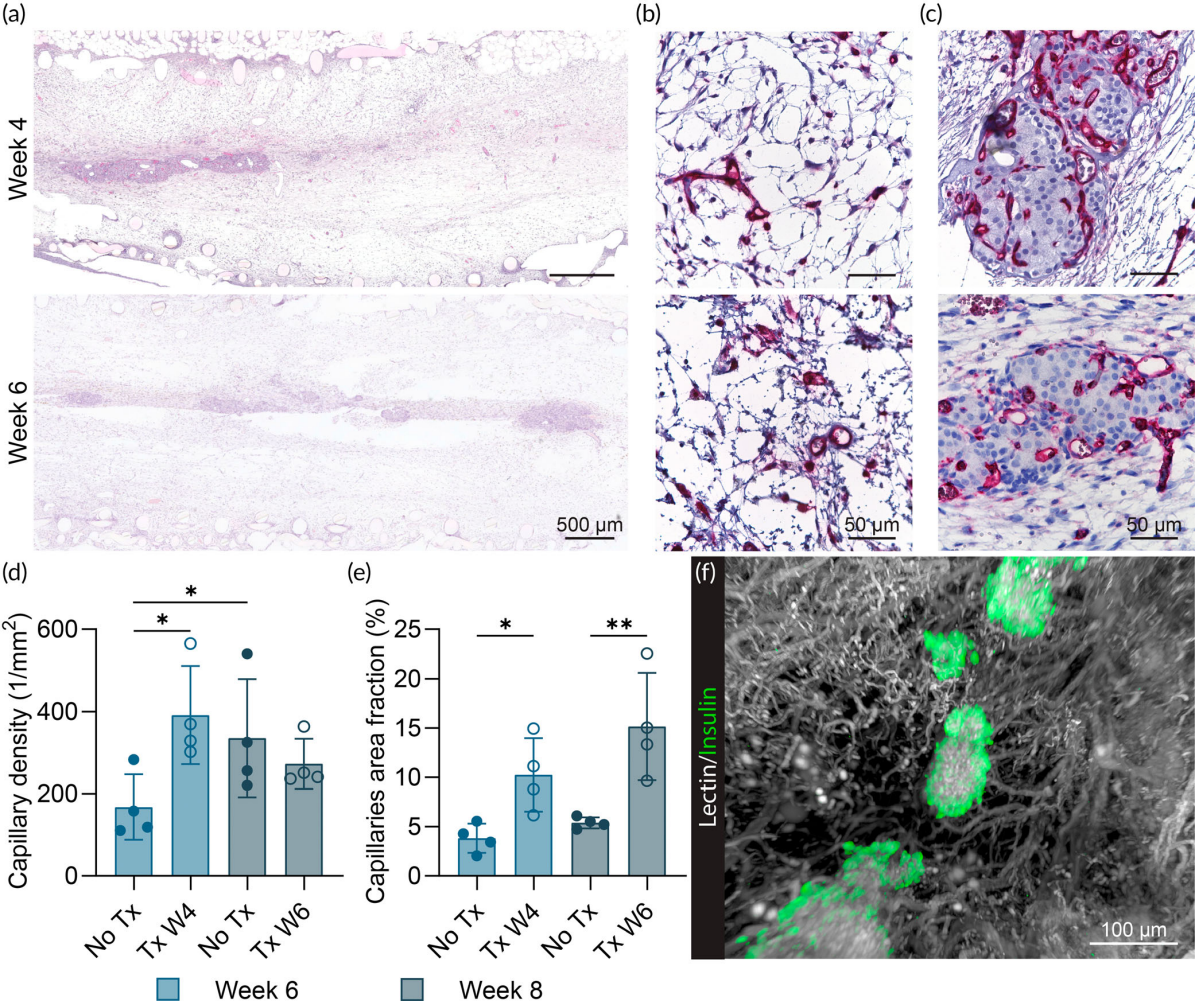
Histological assessment of islets engraftment and revascularization. (a) Representative scans of H&E‐stained sections of NICHE from the groups transplanted at 4 and 6 weeks post‐implantation. Scale bar 500 μm. (b) Representative images acquired at 40× magnification of lectin (pink) stained sections of NICHE cell reservoir tissue from the groups transplanted at 4 and 6 weeks post‐implantation and explanted 2 weeks post‐transplantation. Scale bar 50 μm. (c) Representative images acquired at 40× magnification of lectin (pink) stained sections of pancreatic islets 2 weeks post‐transplantation in NICHE devices implanted for 4 and 6 weeks. Scale bar 50 μm. (d) Capillary density and (e) capillaries fractional area obtained from images of lectin‐stained sections (*n* = 4/group). (f) Lightsheet microscopy 3D image of revascularized pancreatic islets transplanted in NICHE cell reservoir tissue stained with lectin (white) and insulin (green). Scale bar 100 μm. One‐way ANOVA *p < 0.05, **p < 0.01.

Histological analysis was performed on longitudinal sections of the NICHE devices explanted 2 weeks post‐transplantation. H&E scans revealed pancreatic islets integrated within the vascularized cell reservoir tissue (Figure 5a). The islets were aligned within the tissue as a consequence of the delivery strategy, which entails the advancement of a needle through the central silicon port until the opposite end of the cell reservoir. As the needle is retracted, pancreatic islet suspended in a collagen matrix are dispensed, aligning along the needle track.^53^

A dense vascular network was observed on lectin‐stained sections of the cell reservoir (Figure 5b) in both groups. Furthermore, extensive vascularization formed within the transplanted islets (Figures 5c and S1), leading to an overall increase in vascular density compared to non‐transplanted devices (No Tx) that were implanted for the same duration (Figure 5d,e). Lightsheet microscopy 3D dataset analysis further confirmed the revascularization of the pancreatic islets (Figure 5f). These histological evaluations are in line with the increase in T2 values observed 2 weeks post‐islet transplantation (Figures 4c,d and S2).

Taken together, these results highlight the potential of non‐contrast T2 mapping as an estimate for neovascularization and a valuable indicator for assessing the microenvironment of cell encapsulation platforms prior to transplantation. A limitation of this non‐contrast T2 mapping method is the signal contribution from oxygen saturation, however. Future work will build upon the vascular analysis performed in this study to determine oxygenation changes within the device. While contrast‐enhanced MRI has been employed in other studies to assess the vascularization of a subcutaneous microenvironment for cell transplantation,^54^ our approach can achieve similar results without the need of a contrast agent. This non‐invasive approach could enable personalized optimization of the timing of islet transplantation, leading to improved engraftment and function which translates in improved therapeutic outcomes.

## 4 CONCLUSION

In this study we utilized two imaging modalities, nCECT and T2 mapping via MRI, to evaluate vascularization of a cell encapsulation device with direct vascularization. nCECT enabled longitudinal tracking of blood vessels and capillaries ingrowth within our platform, providing anatomical insights into their origin and development. In vivo FBV measurements obtained via nCECT correlated well with ex vivo histological analyses of vascularization.

Relative neovascularization changes within the device were estimated via T2 mapping MRI, both before and after pancreatic islet transplantation performed at different stages of the platform's vascularization. Our results indicate a decrease in T2 values within the device microenvironment 1‐day post islet transplantation, followed by a significant increase, due to local tissue microenvironment remodeling and islet revascularization. Notably, T2 values and vascularization measurements obtained via nCECT and MRI, respectively, showed a strong correlation. This suggests that MRI, which is generally safer than CT, might be sufficient to provide comprehensive information about the transplantation site readiness and suitability to support viability of transplanted cells, without the use of a contrast agent. In addition, this approach can be applied to monitor the status of the graft microenvironment post‐transplantation, as long‐term function of pancreatic islets is often impaired by chronic fibrosis which limits oxygen delivery.^10^, ^55^

Overall, we validated non‐invasive in vivo approaches to assess the readiness of a vascularized microenvironment for cell transplantation. These methods can facilitate precise, personalized timing for the transplantation and facile post‐transplant microenvironment monitoring, potentially increasing the success rate and therapeutic outcomes of the procedure.

## AUTHOR CONTRIBUTIONS

SC: Conceptualization, Methodology, Validation, Formal analysis, Investigation, Data curation, Writing—Original Draft, Writing—Review & Editing, Visualization; JNCC: Methodology, Investigation; NH: Methodology, Investigation; RTRM: Investigation; RB: Methodology, Investigation, Resources; GER: Methodology, Investigation; LD: Methodology, Investigation; KBG: Methodology, Formal analysis, Resources; AVA: Resources, Supervision, Writing—Review & Editing; CYXC: Validation, Writing—Review & Editing; AAB: Conceptualization, Methodology, Validation, Formal analysis, Investigation, Resources, Data curation, Writing—Review & Editing, Visualization, Supervision, Funding acquisition; AG: Conceptualization, Validation, Writing—Review & Editing, Supervision, Project administration, Funding acquisition.

## FUNDING INFORMATION

Funding support from JDRF 2‐SRA‐2021‐1078‐S‐B (AG, AAB), Vivian Smith Foundation (AG), Men of Distinction (AG, CYXC).

## CONFLICT OF INTEREST STATEMENT

AG, SC, and CYXC are inventors of intellectual property licensed by Continuity Biosciences. The other authors declare no conflicts of interest.

## ETHICS STATEMENT

The care and use of the animals employed in this research adhered to the guidelines set by the American Association for Laboratory Animal Science. The rats were housed at the Houston Methodist Research Institute (HMRI) animal facility and the Texas Children's Hospital Feigin Center animal vivarium, with the research activities taking place under the auspices of the HMRI Comparative Medicine Program (CMP). These activities were conducted in compliance with the Animal Welfare Act, the Public Health Service (PHS) Animal Welfare Policy, and the NIH Guide for the Care and Use of Laboratory Animals' principles. The Institutional Animal Care and Use Committee (IACUC) at HMRI and Baylor College of Medicine. approved the humane use of animals in this research, as well as all procedures outlined in the IACUC protocol number IS00007362 (HMRI) and AN‐8907 (BCM).

## Supporting information


**FIGURE S1:** Intra‐islet revascularization. (a) Capillary density and (b) capillary area fraction within pancreatic islets two weeks post transplantation (*n* = 4/group).
**FIGURE S2:** Average T2 values calculated within NICHE cell reservoir (*n* = 3/group).

## Data Availability

The data that support the findings of this study are available from the corresponding author upon reasonable request.
