# Supplementary material for: Nanoparticle contrast‐enhanced computed tomography and magnetic resonance imaging of vascularization of a subcutaneous niche for islet transplantation
Source: Bioeng Transl Med. 2024 Dec 13;10(3):e10740. doi: 10.1002/btm2.10740 (PMC12079526; doi:10.1002/btm2.10740)
Supplement: Supplementary file 1 — FIGURE S1: Intra‐islet revascularization. (a) Capillary density and (b) capillary area fraction within pancreatic islets two weeks post transplantation (n = 4/group). FIGURE S2: Average T2 values calculated within NICHE cell reservoir (n = 3/group). [file BTM2-10-e10740-s001.docx]

Nanoparticle contrast-enhanced computed tomography and magnetic resonance imaging of vascularization of a subcutaneous niche for islet transplantation

Simone Capuani, Jocelyn Nikita Campa-Carranza, Nathanael Hernandez, Renuka T. R. Menon, Rohan Bhavane, Gabrielle E. Rome, Laxman Devkota, Ketan B. Ghaghada, Ananth V. Annapragada, Corrine Ying Xuan Chua, Andrew A. Badachhape, Alessandro Grattoni

Supplementary information


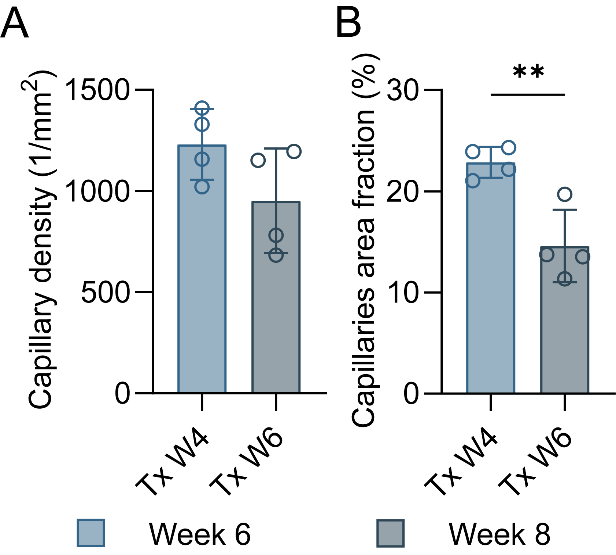


Figure S1. Intra-islet revascularization. A) Capillary density and B) capillary area fraction within pancreatic islets two weeks post transplantation (n=4/group).


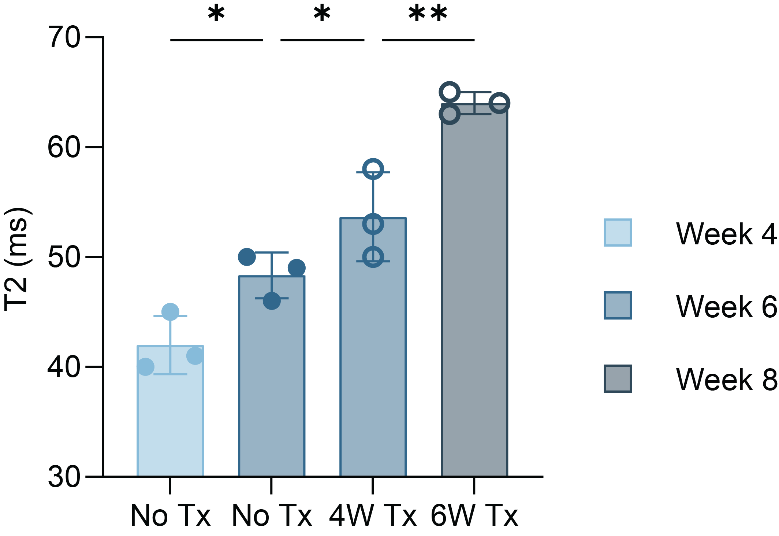


Figure S2. Average T2 values calculated within NICHE cell reservoir (n=3/group)
